# Editorial: 30th anniversary of the molecular cloning and identification of the Na-Cl cotransporter, NCC

**DOI:** 10.3389/fphys.2023.1221806

**Published:** 2023-06-07

**Authors:** Paola de los Heros, David H. Ellison, Gerardo Gamba

**Affiliations:** ^1^ Unidad UNAM-INC, División de Investigación, Facultad de Medicina, Universidad Nacional Autónoma de México, Mexico City, Mexico; ^2^ Department of Physiology and Pharmacology, School of Medicine, Oregon Health and Science University, Portland, OR, United States; ^3^ Department of Nephrology and Mineral Metabolism, Instituto Nacional de Ciencias Médicas y Nutrición Salvador Zubirán, Mexico City, Mexico; ^4^ Molecular Phisiology Unit, Instituto de Investigaciones Biomédicas, Universidad Nacional Autónoma de México, Mexico City, Mexico

**Keywords:** NCC, hypertension, SLC12A, WNK kinases, kidney, structure-function, ubiquitin ligase (E3), phosphatases

The present Research Topic includes 6 papers, that aim to review some of the work done characterizing the *SLC12A* family of cation-coupled electroneutral cotransporters since 1993 in which the NaCl cotransporter (NCC) was first identified, and its cDNA was cloned (Gamba et al., 1994). NCC constitutes the main NaCl entry in the distal convoluted tubule where the fine regulation of its activity by posttranslational modifications, hormones and dietary electrolytes is involved in blood pressure regulation and systemic K^+^ homeostasis. With 2 original papers and four reviews this Research Topic covers different aspects around research of the SLC12A family, from the original functional properties that are characteristic of NCC to the kinases and phosphatases that regulate its activity, as well as its interactions with other proteins in the kidney, such as ubiquitin ligases and K^+^ channels.

NCC cloning in 1993 by Gamba and collaborators, opened the door for the future identification of all *SLC12A* family members, i.e., the Na^+^-K^+^-Cl cotransporters, NKCCs and the K^+^-Cl^−^ cotransporters or KCCs. On the following years several contributions reporting the cloning and characterization of the different cotransporters were published (Gamba et al., 1994; Mount et al., 1999). Since then, studies of the *SLC12A* have extended worldwide and continue providing information of their different roles in the cell and the regulation of several physiological processes. For example, in their present review, Talifu et al. analyze the physiology and pathology of NKCC1 and KCC2 on spinal cord injury, showing how intra and extra neuronal Cl^−^ concentration regulation by both cotransporters is involved in central nervous system electrolytes homeostasis. Their report showcases the role of NKCC1 and KCC2 in spinal cord injury, as well as the regulatory mechanisms in the recovery from this disabling disease.

A breakthrough in NCC history occurred when hypertension-causing WNK kinases were linked to NCC function (Wilson et al., 2003). Research in this field established the WNK/SPAK-OSR1 signaling pathway as the main regulatory mechanism for NCC and all the SCL12A members (Vitari et al., 2005). In their present review paper, Uchida et al. reflect on the physiological significance of this regulation particularly on kidney electrolyte homeostasis. They present a dissertation about how the information regarding the WNK modulation of NCC evolved along the years with contributions from many different groups.

Although the phosphorylation process of NCC by the WNK-SPAK pathway has been revealed with certain detail, a less explored area of research is the one related to NCC dephosphorylation by protein phosphatases (PPs). Recent reports have shown that protein phosphatase 1 (PP1), protein phosphatase 2A (PP2A), calcineurin (CN), and protein phosphatase 4 (PP4) are involved in NCC dephosphorylation (Glover et al., 2010; Picard et al., 2014; Shoda et al., 2017). On this Research Topic, Carbajal-Contreras et al. review phosphatase-mediated modulation of NCC and its interactors in some physiological states where NCC activity is fundamental. It is known for instance that the mechanism of the cyclosporine and tacrolimus induce hypertension is in part due to the inhibition of NCC dephosphorylation, promoting its activity and thus the salt reabsorption.

NCC activity has been shown to be affected by many factors, one recently discovered is regulation by extracellular potassium concentration (Hoorn et al., 2020). On the present Research Topic, Rosenbaek et al. analyzed the role of Nedd4-2 on potassium-induced NCC downward expression showing that this E3 ubiquitin ligase is not involved on this process. Nevertheless, lower NCC phosphorylation following high dietary K^+^ intake is due to reduced activity of the inwardly rectifying potassium channels Kir4.1/Kir5.1, changes in the basolateral plasma membrane potential, and reduced activity of the WNK-SPAK kinase signaling pathway. Meng et al. have shown that Kir5.1 interacts with the E3 ubiquitin ligase Nedd4-2, which then regulates Kir4.1 ubiquitination (Wang et al., 2018b). In their original research paper Meng et al. demonstrate that the effect of high-dietary K^+^ on Kir4.1/Kir5.1 and ROMK in the distal convoluted tubule (DCT) is not affected by gender or Cl^−^ content of the diet.

Finally, NCC structure-function relations, as well as kinetics and pharmacological properties, have been analyzed since its identification 30 years ago, providing fundamental information on NCC structure and function (Monroy et al., 2000). New cryo-EM NCC analysis confirmed and delivered new insights of important transport coordinating residues, ion and thiazide inhibitor binding sites and specific cotransporter regions. The finding of the key residues for polythiazide binding to the transporter is an important discovery that will open the possibility to develop newer and more potent thiazide-type diuretics (Nan et al., 2022). In their review Moreno et al. review the functional characterization of different NCC species from the very first identified NCC to the 2023 cryo-EM NCC structure characterization.

NCC research is far from concluded; new perspectives and information around its regulation, mechanisms, pharmacology, and therapeutics continue to raise questions and provide data about this unique NaCl renal cotransporter. In addition, intense research has also been done with other members of the SLC12 family of solute carriers. The function of these important membrane transporters is implicated in cell volume regulation, in the modulation and type of response to neurotransmitters affecting Cl^−^ channels in the postsynaptic membranes and in the transepithelial transport of salt and potassium.
